# Factors Influencing the Decision-Making Process for Undergoing Invasive Prenatal Testing

**DOI:** 10.7759/cureus.58803

**Published:** 2024-04-23

**Authors:** Panagiota Tzela, Panagiotis Antsaklis, Dimitrios Kanellopoulos, Nikolaos Antonakopoulos, Kleanthi Gourounti

**Affiliations:** 1 Department of Midwifery, School of Health and Care Sciences, University of West Attica, Athens, GRC; 2 Department of Obstetrics and Gynecology, National and Kapodistrian University of Athens, Athens, GRC; 3 Department of Obstetrics and Gynecology, School of Health Sciences, University of Patras, Patras, GRC

**Keywords:** healthcare guidance, informed choice, psychological influences, clinical characteristics, demographic factors, decision-making, invasive prenatal testing

## Abstract

Invasive prenatal testing, amniocentesis, and chorionic villus sampling offer insights into fetal genetic integrity and health, but carry inevitable minor risks of miscarriage and infection, thus complicating the decision-making process for parents. Previous research has revealed several factors that influence the decision to undergo invasive prenatal testing, including demographic, clinical, and psychological aspects, and attitudes towards testing. Informed choice, involving understanding options and aligning them with personal values, is crucial, with healthcare providers playing a key role in offering unbiased information. This systematic review aims to gather and synthesize literature data on the above factors to draw conclusions to aid antenatal care providers in supporting couples to make more informed decisions about their prenatal care.

A systematic search was performed in PubMed and PsycInfo databases using the appropriate keywords and an in-depth evaluation of the studies retrieved followed. Finally, 17 articles were eligible for our review investigating the decision-making process of invasive prenatal testing.

Factors like maternal age, education, and ethnicity are pivotal during the decision-making process. Clinical characteristics also influence decisions and women with pregnancies categorized as high-risk or those who have undergone fertility treatment display a preference for invasive testing. There seems to be a direct correlation between a woman's willingness to consider pregnancy termination, deeply rooted in psychological and moral stances, and the inclination to undergo invasive testing. In the patient decision-making process, the provision and depth of knowledge are of paramount importance. A comprehensive understanding facilitates more informed decisions. Finally, attitudes towards termination of pregnancy, as another factor influencing the decision-making process, reveal a nuanced landscape where personal beliefs, religious considerations, legal restrictions, and perspectives on disability converge. Within this complex context, religion emerges as an important determinant, shaping individuals' views on the morality of abortion.

This review sheds light on the most important factors influencing the couples’ consent for invasive prenatal testing. Healthcare professionals must identify which factors are critical in every specific case among several sociodemographic, clinical, emotional, and religious factors. Thus, they will be able to provide balanced and comprehensive information to help couples under this stressful procedure. We advocate for a patient-centered multidisciplinary approach while navigating couples through the intricate landscape of decision-making concerning invasive prenatal testing.

## Introduction and background

In recent decades, prenatal testing has become a common practice in most developed countries, offering to pregnant women and their partners valuable insights into the health of their fetus which also reflects its long-term prognosis. Invasive prenatal testing, encompassing procedures like amniocentesis and chorionic villus sampling (CVS), facilitates the collection of fetal cells or placental for subsequent genetic analysis, usually karyotyping and chromosomal microarray analysis (CMA) [1,2]. However, these invasive tests are associated with potential adverse events, including miscarriage and infection [3], thus rendering the consultation process challenging and the decision-making process complex and potentially stressful for prospective parents.

Extensive research has revealed several factors that influence the decision to undergo invasive prenatal testing, including demographic and clinical characteristics, psychological traits, and the knowledge, attitudes, and personal beliefs of the couple towards prenatal testing [4]. For instance, advanced maternal age, educational attainment, and income have been identified as factors influencing the decision-making process for invasive prenatal testing [4]. Clinical factors, such as the presence of particular risk of chromosomal abnormality during prenatal screening also play a pivotal role in the decision-making process. An increased risk of chromosomal abnormalities is frequently associated with advanced maternal age or abnormal ultrasound findings and may instigate parents towards invasive prenatal testing [5]. Furthermore, parental anxiety levels and perceived control over the decision-making process also contribute to the decision [6]. Finally, knowledge and attitudes regarding invasive prenatal testing, including beliefs about its benefits and risks, significantly impact decision-making [7].

As generally in medicine, during the above procedure, it is crucial to consider the concept of informed choice. Informed choice refers to the process of gathering relevant information about the available options, weighing the potential benefits and risks, and making a decision that aligns with the individual's values and preferences [8]. Information is offered through the respective healthcare specialist or a team of experts in the field. For prenatal testing usually this is done by fetal medicine consultant and/or a clinical geneticist. Studies have demonstrated that when individuals have access to comprehensive and accurate information about invasive prenatal testing, they are better equipped to make informed choices [9].

Healthcare providers play a critical role in facilitating informed choice by ensuring that individuals receive unbiased information about the procedures involved, potential risks and benefits, as well as the limitations of the tests [10]. This includes providing clear explanations and addressing any questions or concerns that individuals may have [11]. Recognizing and respecting individual values and beliefs is also crucial in promoting informed decision-making [12].

Given the complexity involved in making decisions on invasive prenatal testing, our aim was to systematically review the existing literature and consolidate the current evidence on the factors influencing this decision. Through a comprehensive analysis of the available data, this study aims to identify and analyze the diverse demographic, clinical, psychological, and knowledge-related factors that affect the decision making to undergo invasive prenatal testing. By gaining a deeper understanding of the factors contributing to this decision, healthcare providers can adjust their practice and offer enhanced support to pregnant women and their partners, enabling them to make the appropriate informed decisions about their antenatal prenatal care.

## Review

Methods

Search and Literature Review Strategy

**Figure 1:**
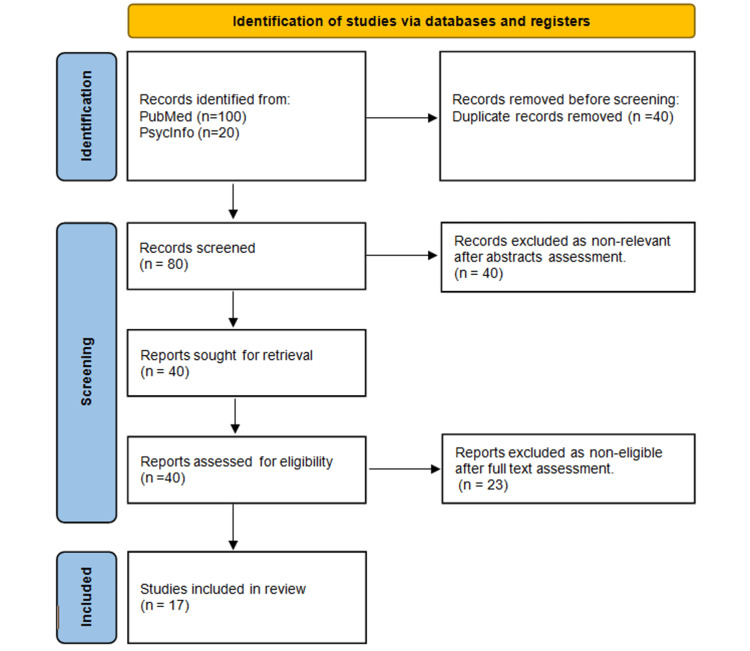
PRISMA diagram of selection process PRISMA: Preferred Reporting Items for Systematic reviews and Meta-Analyses

Our search for potentially relevant articles focused on the PsycInfo and PubMed databases. Our search strategy included a combination of keywords and controlled vocabulary terms related to invasive prenatal testing, patient decision-making, and the relevant factors that we were interested in such as socio-demographic characteristics, clinical characteristics, psychological characteristics, knowledge, personal values and beliefs, and attitudes of couples (Figure 1). We used a combination of free-text terms and Medical Subject Headings (MeSH) terms, and we adapted our search strategy to the specific syntax and indexing conventions of each database.

Inclusion and Exclusion Criteria

To ensure the robustness and reliability of our review, we included only articles presenting original, primary research findings which have been vetted through a peer-review process and then published in highly esteemed journals. Methodologically, we expected these articles to use either quantitative or mixed methods, supporting the research with a solid empirical base. The content of the articles had to explore the decision-making process and the factors influencing it regarding invasive prenatal testing among pregnant women or both parents when applicable.

To maintain the specificity and rigor of our review, we did not embed comments, opinions retrospective studies and literature reviews or articles that were not accessible in their full form. Of particular note is that we specifically excluded research focusing exclusively on non-invasive prenatal testing or research generally on prenatal testing, without making clear distinctions between invasive and non-invasive procedures. Finally, we excluded studies focusing in healthcare professional’s attitudes toward invasive prenatal testing.

Study Selection

Duplication of articles was effectively eliminated by utilizing Zotero software, version 6.0.26 (Corporation for Digital Scholarship, Vienna, VA). During the primary phase of this process, two independent reviewers conducted an initial evaluation of the discovered papers' titles and abstracts. In the subsequent phase, these same researchers engaged in an exhaustive examination of the full texts of all remaining studies, aiming to ascertain their eligibility. In addition, we undertook a thorough examination of the reference lists of both included studies and relevant review articles. Any discrepancies between reviewers were resolved by discussion or by a third reviewer if consensus could not be reached.

Data Extraction

All authors participated in data extraction and analysis. The assemblage of data encapsulated insights derived from both the 'results' and 'discussion' sections of the pertinent studies. The authors assiduously collated data across an array of dimensions: the year of publication, the country of the study, participant demographic information, the risk level of the pregnancy, significant factors which influence of decision-making and finally the methodological soundness of the incorporated studies.

For the purposes of data synthesis, a qualitative assimilation of findings was undertaken. This led to the identification of a multitude of factors integral to the decision-making process regarding invasive prenatal testing. These factors spanned various domains such as sociodemographic and clinical characteristics, test characteristics, personal values and beliefs, psychological facets, attitudes towards pregnancy termination, relational aspects, understanding of invasive prenatal testing, and the provision of information (Table 1).

**Table 1:** Characteristics of included studies

Author, Year	Country	Participants (N)	Risk level of pregnancy	Variables	Significant factors for decision-making
Lewis et al., 2014 [6]	UK	1087 women 18 partners	All risk levels	-Sociodemographic characteristics -Psychosocial factors associated with the decision-making process	Time taken to make a decision about invasive tests
Grinshpun-Cohen et al., 2015 [7]	Israel	42 women	High risk	-Sociodemographic characteristics -Knowledge -Attitude toward pregnancy termination -Psychological factors -Decision-making process	-Age as the main reason for undergoing amniocentesis -Less knowledge about the related risk with amniocentesis -Positive correlation between willingness to consider pregnancy termination and amniocentesis uptake -Want certainty, fear of Down's syndrome
Grinshpun-Cohen et al., 2015 [13]	Israel	42 women	Low risk	-Maternal age -Family history	-Impact of advanced maternal age (>35 years old) on the decision for invasive prenatal testing -46,7% amniocentesis of which 86,7% >35 years old
Bangsgaard and Tabor, 2013 [14]	Denmark	543 women 430 partners	All risk levels	-Sociodemographic characteristics -Informed choice -Satisfaction of decision -Fertility treatment	-Most women and men had high degrees of knowledge (82% and 81%) and positive attitudes regarding risk assessment (97% and 98%), leading to 79% and 80% making an informed choice
Fumagalli et al., 2018 [15]	Italy	448 women	Low Risk	-Sociodemographic characteristics -Perceive risk of having a baby with Down's syndrome -Perceive risk of miscarriage of invasive testing	-Impact of perception of risk 1/200 and procedure-related miscarriage on the decision to undergo invasive prenatal testing
Dicke et al., 2014 [16]	USA	2643 women	All risk levels	-Sociodemographic characteristics	-Association between being non-Caucasian and low education level and negative attitude toward invasive prenatal tests
Farrell et al., 2014 [17]	USA	334 women	All risk levels	-Clinical characteristics	-Likelihood of women with a high risk of chromosomal abnormalities undergoing invasive prenatal testing
Gil et al., 2015 [18]	UK	6782 women	All risk levels	-Sociodemographic characteristics -Clinical characteristics	-Association between being non-Caucasian and negative attitude toward invasive prenatal tests -Avoidance of anxiety of waiting results as a reason to refuse invasive investigations following a screening test -Attitude about pregnancy termination: In high-risk or intermediate-risk group women refused invasive testing if they didn’t consider pregnancy termination
Chan et al., 2014 [19]	China	358 women	All risk levels	-Sociodemographic characteristics	-Preference for invasive prenatal diagnosis among those with lower education levels (secondary education or lower)
Cheng et al., 2018 [20]	China	48 women	High risk	-Sociodemographic characteristics -Anxiety and depression -Pregnancy stress	-Anxiety as a predictor of invasive prenatal test uptake
Lund et al., 2018 [21]	Denmark	315 women 102 partners	All risk levels	-Sociodemographic characteristics -Fertility treatment -Knowledge	-Preference for invasive testing among high-risk couples who have undergone fertility treatment
Grinshpun-Cohen et al., 2015 [22]	Israel	49 women	High risk	Attitude toward pregnancy termination -Phycological factors	-The most worried women about pregnancy outcome and the least concerned about amnio-related risks were the most likely to undergo amniocentesis
Skutilova, 2015 [23]	Czech Republic	271 women	All risk levels	-Sociodemographic characteristics -Clinical characteristics - Knowledge and information provision -Decision-making process -Feelings over time about invasive prenatal testing	-Significant correlation between previous spontaneous abortion and negative feelings about amniocentesis -Role of the gynecologist in providing information about invasive prenatal testing -Decision-making process: the main reason for undergoing invasive tests is to make sure the fetus is healthy
Farrell et al., 2011 [24]	USA	139 women	All risk levels	-Sociodemographic characteristics -Knowledge -Decision-making process -Values and beliefs	-Impact of knowledge levels on decisions about chorionic villus sampling, an invasive prenatal test -Values and beliefs about pregnancy termination and raising a child with Down's syndrome
Ternby et al., 2015 [25]	Sweden	161 women	All risk levels	- Sociodemographic characteristics -Reasons accept/decline invasive testing -Kind of information about invasive testing received -Knowledge	-Reasons for accepting: to be sure of the baby’s health -Reasons for decline: termination of pregnancy in case of positive outcome, risk of miscarriage -Most of the women were >35 years old
van der Steen et al., 2019 [26]	The Netherlands	181 women 5 physicians	High risk	Physicians: Level of information, Women: - Sociodemographic characteristics -Impression of physician preference -Information provided -Anxiety	-Influence by physician’s pre-test counseling on the decision to undergo invasive prenatal testing
van Schendel et al., 2016 [27]	The Netherlands	1091 women	High risk	- Sociodemographic characteristics -Attitude toward pregnancy termination -Informed choice -Decisional conflict -Anxiety	-Attitude toward pregnancy termination is higher in women who choose invasive prenatal testing -The majority of women opting for invasive prenatal testing made a decision that aligns with their personal values

Results

Sociodemographic Characteristics

Sociodemographic characteristics were significant factors across several studies, indicating their strong influence on the decision-making process related to invasive prenatal testing. Specifically, maternal age has been identified as a significant determinant which exhibited a profound effect of advanced maternal age (>35 years) on the choice for invasive prenatal testing. According to the literature among the 46.7% of participants who opted for amniocentesis, a remarkable 86.7% were over 35 years of age [13].

Education levels have been identified as critical variable. An array of studies has indicated a correlation between higher educational attainment and increased utilization of invasive prenatal testing [14,16-18,28,29]. On the other hand, other studies report that invasive testing, as opposed to NIPT, was associated with lower educational background (p=0.023). More specifically, individuals possessing a secondary education or lower were frequently more inclined towards invasive prenatal diagnosis [19,20]. Thus, only 33% of women who exclusively chose invasive prenatal testing had received tertiary education, compared to more than 50% of women in the control group. This inclination is believed to arise due to limited awareness regarding the associated risks of invasive tests.

Ethnicity also carries significant implications. There is evidence showing a propensity among non-Caucasians (Afro-Caribbean) to demonstrate a negative attitude towards both invasive and non-invasive prenatal tests (OR=0.290, p=0.001) [16,18].

Clinical Characteristics

Clinical characteristics also play a pivotal role in the decision to embark on invasive prenatal testing. The women at high risk of chromosomal abnormalities undergoing invasive prenatal testing are substantial [17]. The preference for invasive testing like CVS in 40% of high-risk cases likely reflects its near-absolute diagnostic accuracy, which is crucial for conditions where the risk of chromosomal abnormalities is deemed high based on initial screenings. The number of these cases is underestimated given that women in high or intermediate risk categories often decline invasive testing if they are disinclined to consider the possibility of pregnancy termination. Another study noted that couples categorized as high-risk and who had undergone fertility treatment displayed a preference for invasive testing in comparison with couples who had spontaneous conception, with a statistically significant difference in preferences (0.56 versus 0.10; p<0.05) [21].

Psychological and Emotional Factors

Psychological factors hold substantial weight in the decision-making process regarding invasive prenatal testing. Among them, anxiety is identified as the primary determinant [20]. Another study highlighted the integral role of psychological considerations, determining that women who were most worried about pregnancy outcomes and least concerned about the risks related to amniocentesis were more likely to undergo the procedure [22]. An additional investigation explored how psychosocial variables influence the time women require to make decisions about invasive tests, signifying their relevance in the decision-making timeline [6]. Furthermore, there seems to be a direct correlation between a woman's willingness to consider pregnancy termination, deeply rooted in psychological and moral stances, and the inclination to undergo amniocentesis, which implies that fears, such as the possibility of Down's syndrome, play a critical role in these choices [7].

Level of Knowledge and Provided Information

In the patient decision-making process, the provision and depth of knowledge are of paramount importance. As expected, a comprehensive understanding facilitates more informed decisions [14]. In contrast, the lack of adequate knowledge is a decisive factor in the uptake of procedures [7]. Separate research highlights how previous experiences, such as undergoing fertility treatments, may influence high-risk couples' inclination towards invasive testing [21]. The pivotal role of gynecologists in steering these decisions is also underscored [23], while further investigations highlighted the profound impact of both the quality and nature of information received [24,25]. Moreover, the importance of well-curated information is further emphasized in literature. This way decisions align with individuals' core values, particularly when contemplating potential pregnancy termination [26].

Personal Beliefs and Attitudes Toward Pregnancy Termination

The existing literature highlights the significant influence of attitudes towards pregnancy termination on the uptake of invasive prenatal testing. A positive correlation between the willingness to consider pregnancy termination and the choice of amniocentesis is pinpointed [7]. Similarly, it was observed that women who prioritized the outcomes of their pregnancy, and showed less concern about amniocentesis risks, were more inclined to opt for the procedure [22]. This becomes obvious by the fact that women over 35 years of age irrespective of the risk for Down's syndrome opted for invasive testing (17 out of 30 women). This perspective was further strengthened by data that indicated a stronger preference for invasive prenatal testing among women with heightened termination considerations, with their choices predominantly aligning with their personal values [27]. Additionally, separate research emphasized that individuals in high or intermediate-risk categories showcased a pronounced hesitation towards invasive testing when they wouldn't contemplate termination as a feasible option [18].

Discussion

The aim of this review was to investigate the factors influencing the decision-making process for undergoing invasive prenatal testing. 

Our findings underscore the significant role of advanced maternal age (>35 years) in elective invasive prenatal testing [7,13]. The common knowledge that the possibility of chromosomal abnormalities, increases with maternal age, could be a major factor behind such decision. The role of educational attainment in relation to the decision-making process is paramount and has profound implications. Evidence from our review indicates a dichotomy in which women with advanced educational backgrounds and those with secondary education or less are predisposed to opt for invasive diagnostic procedures. The first category is characterized by a better overall understanding of their available options [14,16-19,28,29]. Conversely, the second category may be influenced by partial or misinformed perspectives on the invasive procedure, such as the risk of miscarriage [19,20]. Distinct patterns were also observed between ethnic groups. In particular, non-Caucasian women (Afro-Caribbean) showed a reluctance toward invasive prenatal testing [16,18]. Such ethnic diversity indicates the presence of embedded cultural values and social influences.

The clinical characteristics of certain patient groups significantly highlight their own importance. This is particularly evident in the case of women at high risk for chromosomal abnormalities, as well as couples who have undergone the complex journey of fertility treatments [17,21]. These groups demonstrate a noticeable preference for invasive testing, highlighting the necessity for personalized informational and psychological support tailored to their unique circumstances. It can be hypothesized that couples who have put their trust in science to have a child they are ready to follow scientific indications for prenatal diagnosis even if there is a minor risk of miscarriage associated with diagnostic procedure. Furthermore, there exists a notable dichotomy in the decision-making process of women classified as high or intermediate risk [17,21]. These women often exhibit reluctance, or even outright refusal, to undergo invasive testing, especially in scenarios where pregnancy termination is not considered as an option [13,22]. Anyway, the interest lies within cases that termination of pregnancy is an option, and this should be addressed first to make clear if consultation could alter the couple’s decision. Otherwise, putting the fetus at risk of miscarriage is not justified as it can be examined soon after birth. In general, this observation warrants a more in-depth exploration into the ways risk perceptions and decision-making processes develop in these specific contexts.

Invasive testing risk can be expressed in terms of both numerical relevance and acceptability. Perceived acceptability seems to affect the interpretation of a given risk more than the numerical relevance of the risk [15]. A woman may consider the 1/200 risk of miscarriage non-negligible but acceptable, and she may decide to undergo invasive testing. In comparison, a woman may consider the 1/350 risk of carrying a foetus with Down's syndrome negligible but unacceptable and consequently may decide to undergo invasive testing. These findings suggest that acceptability should be a key focus in counselling communication.

From the psychological and emotional factors, anxiety unfolds as pivotal in the decision-making process, revealing a delicate balance where emotional, psychological, and factual knowledge intricately weave into decisions. In our study, we have demonstrated that women experiencing elevated levels of anxiety are more inclined to choose invasive prenatal diagnostic procedures. This inclination arises from their desire to obtain comprehensive information regarding the health status of the fetus, despite the associated risk of miscarriage [6,7,20,22]. The pivotal role of psychological and emotional factors is not only salient in the acceptance of invasive testing but also perceptibly intertwined with decision-making timelines, possibly indicating a potential gap where additional psychological support may be harbored to aid in timelier, informed decisions. The involvement of midwives in this decision-making process is crucial. Midwives, with their expertise in providing holistic care, can play a vital role in offering emotional support to women, navigating through the complexities of prenatal screening decisions [30]. Their presence can contribute to a more comprehensive understanding of the emotional and psychological factors influencing a woman's choice, thereby fostering a balanced and informed decision-making process [30]. Collaborative efforts between healthcare professionals, including midwives, obstetricians, fetal medicine doctors, neonatologists, geneticists, and phycologists may enhance the overall support system available to women during this critical phase of decision-making.

Knowledge and information dissemination stand out as critical determinants, emphasizing that while the depth of knowledge plays a quintessential role in bolstering informed decisions, the sources, quality, and alignment of information with personal values emerge as equally crucial [7,14,21]. In the realm of prenatal screening decisions, the notable impact of healthcare professionals, particularly obstetricians and fetal medicine specialists, in steering decisions throws into relief the necessity of ensuring that these professionals are armed with not only accurate and up-to-date information but also the skills to communicate these effectively and empathetically [23-25].

The pivotal role of healthcare providers extends beyond the mere provision of information, as their ability to navigate and address the emotional and psychological aspects of decision-making significantly influences patient choices [23]. This underscores the importance of integrating psychological training into the education and professional development of healthcare professionals involved in prenatal care. Furthermore, the establishment of interdisciplinary collaboration, including midwives, can contribute significantly to enhancing the holistic support provided to women during this critical decision-making phase [30]. These collaborative efforts not only broaden the spectrum of emotional support available but also reinforce the collaborative nature of healthcare delivery, promoting a more patient-centered approach.

Attitudes towards termination of pregnancy, as another factor influencing the decision-making process, reveal a nuanced landscape where personal beliefs, religious considerations, legal restrictions, and perspectives on disability converge. Within this complex context, religion emerges as an important determinant, shaping individuals' views on the morality of abortion. For instance, having a strong religious faith, particularly in belief systems like Islam countries that vehemently denounce termination of pregnancy, coupled with residing in countries where abortion is prohibited by law, significantly impacts women's decisions to abstain from any form of prenatal testing [31].

Personal beliefs aside, the legal landscape further complicates the discourse. The existence of legal frameworks that restrict or prohibit termination of pregnancy introduces a level of complexity, potentially affecting not only the availability of abortion services but also the social perception of the procedure [31,32]. Considering the specific legal restrictions and their impact on reproductive rights provides essential context for understanding the broader dynamics surrounding termination of pregnancy. Furthermore, delving into the realm of attitudes towards disability adds another layer of complexity. Attitudes toward life and disability contribute significantly to ethical considerations regarding decisions related to invasive prenatal testing. Individuals or couples may face complex ethical dilemmas when faced with the prospect of potential disabilities in the unborn child, further emphasizing the deeply personal nature of these decisions [33].

Tying these multifaceted elements together, it becomes apparent that the moral and ethical compass guiding decisions about invasive prenatal testing is deeply shaped by a combination of personal beliefs, religious values, legal constraints, and disability perspectives. Recognizing this complex interplay highlights the importance of adopting a counseling and decision support approach that is not only respectful of individual beliefs but also ethically and morally sensitive to the various factors that influence the decision-making process regarding pregnancy termination.

## Conclusions

In conclusion, this systematic review provides a comprehensive exploration of the multiple factors influencing the decision-making process for undergoing invasive prenatal testing. The findings have revealed a complex interplay of demographic, clinical, psychological, informational, and attitudinal factors shaping choices individuals and couples make in this critical aspect of reproductive healthcare. In light these findings, it is evident that a holistic and patient-centered approach, involving interdisciplinary collaboration and comprehensive support systems, is essential in navigating the intricate landscape of decision-making surrounding invasive prenatal testing. During this process healthcare professionals should make clear the indications and risks to the couples and finally respect their decisions. That said tailored interventions and policies aiming to promoted informed, balanced, and ethically sensitive choices in prenatal testing should be developed.
